# Supplementary material for: Influence of Anesthesia and Clinical Variables on the Firing Rate, Coefficient of Variation and Multi-Unit Activity of the Subthalamic Nucleus in Patients with Parkinson’s Disease
Source: J Clin Med. 2020 Apr 24;9(4):1229. doi: 10.3390/jcm9041229 (PMC7230272; doi:10.3390/jcm9041229)
Supplement: Supplementary file 1 [file jcm-09-01229-s001.pdf]

Supplementary material 1.

Mixed effect linear regression model for firing rate, CV and MUA. In the analysis, we included demographic and clinical variables as in the standard linear regression model. We added a random effect grouped by patient because our data included multiple observations within each patient. Estimate indicate the slope of the line, when negative indicates decrease and positive indicates increase. * ipsilateral to body side with onset of disease. No changes of the significant values were observed compared to the standard linear regression model.

Variable		Firing rate		Coefficient of variation		Multi-unit activity	
		Estimate	P-value	Estimate	P-value	Estimate	P-value
DEX	Yes	-3.229	0.1072	0.3061	0.0018	-91.494	0.0068
REMI	Yes	-2.011	0.2239	0.1603	0.0493	4.664	0.8636
CLONI	Yes	-2.017	0.3026	-0.0098	0.9192	-52.976	0.0991
PSA DISCONT.	Yes	-3.677	0.1935	-0.0164	0.9064	-111.180	0.0173
SEX	Male	0.479	0.7926	-0.0278	0.7550	-0.755	0.9804
ONSET SIDE	Ipsilateral(*)	-1.932	0.1306	0.0970	0.1688	-16.648	0.0751
HEMISPHERE	Right	1.612	0.2076	-0.0811	0.2513	21.994	0.0184
AGE	Years	-0.067	0.5167	0.0013	0.7958	-3.847	0.0202
WEIGHT	Kg	-0.001	0.9820	-0.0025	0.3680	0.290	0.7491
UPDRS III		0.060	0.3266	0.0022	0.4686	0.074	0.9416
DISEASE DURATION	Months	-0.000	0.9964	-0.0020	0.0241	-0.042	0.8876
STN DEPTH	mm	0.604	0.1005	-0.0081	0.6896	24.009	<0.001
**Model data**		R squared 0.0532<br>Adjusted R-Squared 0.0335		R squared 0.0530<br>Adjusted R-Squared 0.0333		R squared 0.1497<br>Adjusted R-Squared 0.1472	

Table 1 , random effects modeling with patient as a random variable

Supplementary material 2. Mixed effect linear regression model for firing rate and CV for sorters MR, RB and MB.

To test whether our findings of single unit behavior were robust to the subjective nature of manually selecting spike waveforms, we included a mixed effect analysis, grouping data by which author sorted the data (the 'sorter.). In the analysis, we included demographic and clinical variables as in the standard linear regression model. Estimate indicate the slope of the line, when negative indicates decrease and positive indicates increase. * ipsilateral to body side with onset of disease. All the main effects of the standard linear regression with sorter 1 remain in this model.

Variable		Firing rate		Coefficient of variation	
		Estimate	P-value	Estimate	P-value
DEX	Yes	-5.649	0.0007	0.593	0.0306
REMI	Yes	-1.466	0.2484	0.654	0.0019
CLONI	Yes	-1.322	0.3872	0.211	0.4031
PSA DISCONT.	Yes	-3.220	0.1028	0.369	0.2583
SEX	Male	0.545	0.6817	0.027	0.9022
ONSET SIDE	Ipsilateral(*)	1.348	0.2260	-0.481	0.0091
HEMISPHERE	Right	1.308	0.2451	-0.239	0.1990
AGE	Years	0.073	0.3287	0.005	0.6771
WEIGHT	Kg	0.021	0.6054	0.009	0.1933
UPDRS III		-0.109	0.0192	0.017	0.0257
DISEASE DURATION	Months	-0.025	0.0923	0.003	0.1597
STN DEPTH	mm	0.400	0.2053	0.026	0.6216
Model data		R squared	0.0786	R squared	0.0906
		Adjusted R squared	0.0605	Adjusted R squared	0.0728

Table 2, *random effects modeling with sorter as a random variable*
